# Abstract of Meteorological Observations for September, 1855, Made at Philadelphia, Pa.

**Published:** 1855-11

**Authors:** James A. Kirkpatrick


*Abstract of Meteorological Observations for September,* 1855, *made at Philadelphia, Pa. Latitude* 39° 57′ 28″ *N., Longitude* 75° 10′ 40″ *W. from Greenwich.* By PROF. JAMES A. KIRKPATRICK.

| 1855. September. | Barometer. Daily Mean | Barometer. Mean Daily Range. | Thermom. Daily Mean | Thermom. Mean Daily Range | Dew Point 2 P.M. | Force of Vapor 2 P.M. | Rel. Humid. 2 P.M. | Rain. | Prevailing Winds. | Remarks. |
|---|---|---|---|---|---|---|---|---|---|---|
| | Inches. | Inches. | Deg. | Deg. | Deg. | Inches. | Hunds. | Inch. | Points. | |
| 1 | 30.104 | .125 | 76.8 | 9.2 | 71.4 | .767 | .73 | | SW. | M. cl'dy; aft. and ev. clear. |
| 2 | 29.924 | .180 | 80.5 | 3.7 | 71.9 | .781 | .66 | | SW. | Clear. |
| 3 | 29.952 | .037 | 70.0 | 10.5 | 61.5 | .546 | .67 | } 0.685 | NE. | Cloudy; aft. and ev. *rain.* |
| 4 | 30.060 | .109 | 65.7 | 4.3 | 61.6 | .549 | .89 | | NE. | M. and aft. *rain,* ev. clear. |
| 5 | 30.096 | .195 | 66.8 | 4.2 | 53.4 | .409 | .50 | | (Var.) | Clear. |
| 6 | 30.181 | .103 | 67.0 | 3.2 | 56.0 | .450 | .52 | | (Var.) | Clear. |
| 7 | 30.142 | .037 | 69.0 | 2.7 | 59.8 | .515 | .61 | | SW. | M. fog: aft. and ev. clear. |
| 8 | 29.980 | .162 | 77.2 | 8.2 | 63.2 | .570 | .47 | | SW. | Clear. |
| 9 | 29.894 | .086 | 81.3 | 4.3 | 70 5 | .746 | .58 | | W. | Clear. |
| 10 | 29.896 | .024 | 78.3 | 3.5 | 68.2 | .689 | .57 | | W. | Clear. |
| 11 | 29.922 | .026 | 81.0 | 3.3 | 68.8 | .704 | .55 | | SW. | Clear. |
| 12 | 29.894 | .053 | 83.7 | 2.7 | 70.6 | .749 | .53 | | SW. | Clear. *Therm. highest* 90° |
| 13 | 29.853 | .078 | 80.2 | 5.2 | 70.5 | .746 | .58 | 0.317 | (Var.) | M. cl'dy, aft. *rain,* ev. clear. |
| 14 | 30.047 | .194 | 69.2 | 11.0 | 54.8 | .429 | .51 | | NE. | Cloudy. |
| 15 | 29.996 | .051 | 70.7 | 2.2 | 65.0 | .618 | .76 | 0.048 | E. | M. & aft. *rain,* ev. clear. |
| 16 | 29.866 | .131 | 72.3 | 1.7 | 69.9 | .730 | .81 | 0.090 | NW. | M. *rain,* aft. cl'y, ev. clear. |
| 17 | 29.894 | .029 | 77.3 | 5.0 | 65.3 | .624 | .58 | | SW. | M. cloudy, aft. & ev. clear. |
| 18 | 29.705 | .223 | 80.8 | 3.5 | 69.4 | .717 | .58 | | SW. | Cl'dy. *Bar. lowest* 29.623. |
| 19 | 30.102 | .397 | 59.3 | 21.5 | 43.9 | .287 | .45 | 0.400 | NE. | M. *rain,* aft. and ev. clear. *Therm. lowest,* 45° |
| 20 | 30.171 | .103 | 58.8 | 0.8 | 48.8 | .345 | .54 | | NE. | M. clear; aft. and ev. cl'dy. *Barom. highest* 30.224. |
| 21 | 29.970 | .201 | 66.3 | 7.5 | 63.2 | .578 | .85 | 0.438 | SW. | M. cl'y; aft. ev. & n't. *rain.* |
| 22 | 29.859 | .117 | 68.0 | 2.3 | 64.0 | .595 | .76 | | (Var.) | M. and aft. cl'dy; ev. clear. |
| 23 | 30.102 | .244 | 61.3 | 6.7 | 51.0 | .375 | .59 | | NE. | M. clear; aft. & ev. cloudy. |
| 24 | 30.193 | .057 | 60 2 | 2.2 | 49.6 | .355 | .53 | | NE. | Clear. |
| 25 | 30.023 | .170 | 63.7 | 3.5 | 58.4 | .490 | .63 | | SW. | Clear |
| 26 | 29.747 | .276 | 70.3 | 6.7 | 62.4 | .564 | .61 | 0.686 | SW. | M. & aft. cl'r; ev. & n't. *rain.* |
| 27 | 29.808 | .157 | 64.2 | 7.8 | 49.4 | .353 | .48 | | NW. | M. cl'dy, aft. and ev. clear. |
| 28 | 30.062 | .254 | 59.5 | 4.7 | 45.1 | .300 | .49 | | NW. | Clear. |
| 29 | 30.100 | .042 | 60.5 | 4.3 | 50.8 | .372 | .49 | | (Var.) | Clear. |
| 30 | 29.916 | .184 | 66.0 | 5.5 | 63.3 | .581 | .71 | 1.465 | NE. | Cl'dy; aft. ev. & night *rain.* |
| Means for Sept., 1855 | 29.982 | .135 | 70.2 | 5.4 | 60.7 | .551 | .61 | 4.129 | N. 67° W. 17-100. | |
| Means for Sept., 5 yrs. | 30.009 | .132 | 68.4 | 5.7 | 54.3 | | 61. | 3.690 | West 36-100. | |

The Monthly Range of the Mercury in the Barometer was 0.601 of an inch, and in the Thermometer 45°.

